# Connexin30-Deficiency Causes Mild Hearing Loss With the Reduction of Endocochlear Potential and ATP Release

**DOI:** 10.3389/fncel.2021.819194

**Published:** 2022-01-17

**Authors:** Junmin Chen, Penghui Chen, Baihui He, Tianyu Gong, Yue Li, Jifang Zhang, Jingrong Lv, Fabio Mammano, Shule Hou, Jun Yang

**Affiliations:** ^1^Department of Otorhinolaryngology—Head & Neck Surgery, Xinhua Hospital, Shanghai Jiaotong University School of Medicine, Shanghai, China; ^2^Ear Institute, Shanghai Jiaotong University School of Medicine, Shanghai, China; ^3^Shanghai Key Laboratory of Translational Medicine on Ear and Nose Diseases, Shanghai, China; ^4^Department of Physics and Astronomy “G. Galilei”, University of Padua, Padua, Italy; ^5^Department of Biomedical Sciences, Institute of Cell Biology and Neurobiology, Italian National Research Council, Monterotondo, Italy

**Keywords:** connexin30, hearing loss, connexin26, stria vascularis, endocochlear potential

## Abstract

*GJB2* and *GJB6* are adjacent genes encoding connexin 26 (Cx26) and connexin 30 (Cx30), respectively, with overlapping expressions in the inner ear. Both genes are associated with the commonest monogenic hearing disorder, recessive isolated deafness DFNB1. Cx26 plays an important role in auditory development, while the role of Cx30 in hearing remains controversial. Previous studies found that Cx30 knockout mice had severe hearing loss along with a 90% reduction in Cx26, while another Cx30 knockout mouse model showed normal hearing with nearly half of Cx26 preserved. In this study, we used CRISPR/Cas9 technology to establish a new Cx30 knockout mouse model (Cx30^−/−^), which preserves approximately 70% of Cx26. We found that the 1, 3, and 6-month-old Cx30^−/−^ mice showed mild hearing loss at full frequency. Immunofluorescence and HE staining suggested no significant differences in microstructure of the cochlea between Cx30^−/−^ mice and wild-type mice. However, transmission electron microscopy showed slight cavity-like damage in the stria vascularis of Cx30^−/−^ mice. And Cx30 deficiency reduced the production of endocochlear potential (EP) and the release of ATP, which may have induced hearing loss. Taken together, this study showed that lack of Cx30 can lead to hearing loss with an approximately 30% reduction of Cx26 in the present Cx30 knockout model. Hence, Cx30 may play an important rather than redundant role in hearing development.

## Introduction

Hearing loss is a major global health problem, which can be caused by continued excessive noise (Guo et al., 2021), ototoxic drugs (Breglio et al., 2020), aging (Guo et al., 2021), genetic factors (Fu et al., 2021), and infections (Zhang et al., 2021), among which genetic factors caused 50% of the hearing loss. *GJB2* and *GJB6* are two adjacent genes encoding connexin 26 (Cx26) and connexin 30 (Cx30) respectively which form gap junctions (GJs) between supporting cells (SCs) or exist as the hemichannels at the surface of the SCs in the cochlea (Mammano, 2019). There are two independent GJ networks identified in the inner ear: the epithelial gap junctional network in the organ of Corti and the connective tissue gap junctional network in the cochlear lateral wall (Kikuchi et al., 1995).

Auditory development depends not only on the functional maturation of cochlear hair cells (HCs) but also on the normal differentiation and organization of nonsensory SCs. SCs are coupled through Cx26 and/or Cx30 GJ channels or hemichannels to form a supporting cell network, transmit ATP, ions, signals, and nutrient molecules (Bruzzone et al., 1996; Jagger and Forge, 2015; Verselis, 2019), and the microenvironment of surrounding HCs (Chen et al., 2018). ATP triggers cytosolic Ca^2+^ concentration oscillations and propagation of intercellular Ca^2+^ waves, which appear to play a crucial role in the normal development of the cochlear sensory epithelium, hearing acquisition and the functional maturation of HCs (Johnson et al., 2017; Mammano and Bortolozzi, 2018; Mazzarda et al., 2020). Chai et al. (He et al., 2017; Zhang et al., 2020a, b; Zhang Y. et al., 2020) further confirmed that regenerating HCs cannot achieve functional maturity in the absence of a normal and stable microenvironment by SCs.

*GJB2* and *GJB6* mutations account for up to 50% of all nonsyndromic hearing loss cases (Denoyelle et al., 1997; Estivill et al., 1998; Mishra et al., 2018). Homozygous Cx26 and Cx30 deletions lead to severe hearing loss in animal models (Kelsell et al., 1997; del Castillo et al., 2002). It has been reported that Cx26 and Cx30 are both important for hearing development (Adadey et al., 2020). Notably, Cx30 knockout mice displayed severe hearing loss in the absence of endocochlear potential, accompanied by a 90% reduction in Cx26 (Teubner et al., 2003; Boulay et al., 2013). Over-expression of Cx26 in Cx30 knockout mice rescued hearing (Ahmad et al., 2007), but not *vice versa* (Qu et al., 2012). These results indicated that Cx26 is critical for hearing development since it could not be substituted by Cx30 in early cochlear development. Cx30-deficient mice with nearly 50% Cx26 expression showed normal hearing (Boulay et al., 2013). Therefore, the role of Cx30 in the cochlea remains controversial.

In this study, we used CRISPR/Cas9 technology to establish a novel Cx30 knockout mouse model (Cx30^−/−^), which preserved approximately 70% of Cx26 expression in the cochlea and displayed mild hearing loss under full frequency. This new genotype model showed different morphological and functional phenotypes from the past. We believe that this model is of significant importance to explore the mechanism of hearing loss caused by Cx30 deletion.

## Materials and Methods

### Creation and Genotyping of Cx30 Knockout Mice

Cx30 knockout mice (Cx30^−/−^) maintained on a pure C57BL/6 background were purchased from the Shanghai Model Organisms. CRISPR/Cas9 technology was used to repair the mutation introduced by non-homologous recombination, resulting in the shift of the protein reading frame of the *Gjb6* Exon3 gene and loss of function. The mouse genotyping was identified by PCR amplification with the following primers: *Gjb6* primer 1 (P1) 5’-CTAGTGCAACAGCACCCGTA-3’, *Gjb6* P2 5’-CTAGTGCTGAAGGTGTGGGG-3’,*Gjb6* P3 5’-TGGCATTGTTTCACCGTAGT-3’, *Gjb*6 P4 5’-AGGTCATGTGAATCTGTCTC-3’, Wild type (WT): *Gjb6* P1 and P2 PCR to obtain a single 2369 bp band; *Gjb6* P3 and P4 can obtain a 512 bp band; Heterozygous (HE): *Gjb6* P1 and P2 PCR to obtain both 527 bp and 2,369 bp bands; *Gjb6* P3 and P4 to obtain 512 bp band; Homozygous (HO): *Gjb6* P1 and P2 PCR obtained a single 527 bp band; *Gjb6* P3 and P4 could not obtain a band. The WT littermates served as controls in the experiment. The experimental procedures were approved by the Shanghai Xinhua Hospital’s Animal Center and conducted according to the standards of the NIH Guidelines for the Care and Use of Laboratory Animals.

### Assessment of Hearing Loss

Auditory brainstem response (ABR) reflects the electrical response of the cochlear ganglion neurons and the nuclei of the central auditory pathway to sound stimulation. Their threshold assesses the cochlear sensitivity and it is visually measured according to the occurrence of Wave II in a series of repeatable ABR responses obtained at various sound intensities. After 1, 3, and 6-month-old littermates were anesthetized with ketamine (80 mg/kg) and xylazine (10 mg/kg), they were subjected to sudden tones of various frequencies from 4 to 32 kHz (duration of 10 ms, rising -The fall time is 0.5 ms). The sound is generated by Tucker-Davis System II hardware and software (Tucker-Davis Technologies, Alachua, FL).

### Quantitative PCR Analyses

Total RNA was extracted from the whole dissected cochlea, the basemental membrane (BM), or the stria vascularis (SV) of postnatal day 5 (P5, where P0 is the date of birth) and 1-month-old mice using the Takara MiniBEST Universal RNA Extraction Kit (Takara, 9767, Japan). Reverse transcription was performed using 1 μg of RNA. Quantitative PCR (qPCR) was conducted on cDNAs using. The primers used are as follows:

Cx30 primers: Forward 5’-AAGACCTGGAGGACATCAAACG-3’ and

Reverse 5’-CGAAATGAAGCAGTCCACGAG-3’

Cx26 primers: Foward 5’-TCACAGAGCTGTGCTATTTG-3’ and

Reverse 5’-ACTGGTCTTTTGGACTTTCC-3’

Actin primers: Foward 5’-GAGAGGGAAATCGTGCGTGA-3’ and

Reverse 5’-ACATCTGCTGGAAGGTGGAC-3’),

using TB Green^®^ Premix Ex Taq^TM^ (Takara, RR420B, Japan). Samples were analyzed in triplicate on Applied Biosystems 7500 (ThermoFisher). The expression levels of mRNAs were calculated by the 2^−ΔΔCT^ method.

### Western Blot Analysis

Proteins were extracted from the whole dissected cochlea, the BM, or the SV of P5 and 1-month-old mice. The proteins were separated by SDS-PAGE and transferred to PVDF membranes (Millipore, Billerica, MA, USA). The membranes were blocked with 5% skim milk (Beyotime, Shanghai, China) at room temperature for 1 h and then incubated with primary antibodies against Tublin (Zen Bioscience, 330628, 1:1,000, China), Cx30 antibody (Invitrogen, 71-2800, 1:200, USA), and Cx26 antibody (Invitrogen, 33-5800, 1:200, USA) at 4°C overnight. After three times of washing with PBS-0.01% Tween 20 (PBS-T), the membranes were incubated with a secondary antibody, anti-rabbit IgG, or antimouse IgG (Beyotime; 1:1,000, China), for 2 h at 37°C. After washing the membranes, and adding freshly prepared chemiluminescence solution (Millipore; A:B = 1:1), the immunoreactive bands were imaged under the Bio-Rad ChemiDoc XRS+ (Bio-Rad Co., Hercules, CA, USA). Semiquantitative densitometric analysis was performed with ImageJ software.

### Immunohistochemistry and Confocal Imaging

Cochleas of P5 and 1-month-old mice were fixed with 4% paraformaldehyde, decalcified, frozen, and cut by a cryostat. The tissue sections were directly mounted onto glass slides for staining and storage. The cochlear section was incubated in a blocking solution (10% goat serum and 1% BSA in the PBS) with 0.1% Triton X-100 for 1 h at room temperature. Then, the section was incubated with rabbit anti-Cx30 antibody (Invitrogen, 71-2800, 1:200, USA), the rabbit anti-Cx26 antibody (Invitrogen, 51-2800, 1:200, USA), or the mouse anti-Cx26 antibody (Invitrogen, 33-5800, 1:200, USA) in the blocking solution at 4°C overnight, following reaction with corresponding Alexa Fluor 488-, 458- or 647- secondary antibodies (Invitrogen, 1:500, USA) for 2 h at room temperature (23°C). The sections were further stained by 1% 40, 6-diamidino-2-phenylindole (DAPI, Invitrogen, 1:200, USA) for ~10 min or AlexaFluor 568 phalloidin (Servicebio, G1041, 1:500) for ~15 min following the 2nd antibody incubation to visualize cell nuclei or F–Actin. After washout, the sections were mounted and observed under a microscope.

### ATP Release Measurement

The P5 mouse temporal bone was micro-dissected in ice-cold HBSS (Thermo Fisher Scientific). The inner ear was opened from its apex to base. After removal of the bone, the exposed BM and SV were dissected separately and put into an incubation chamber. For testing ATP release, the isolated BM and SV was incubated in a zero Ca^2+^ solution (ZCS) containing (in mM): 137 NaCl, 5.36 KCl, 0.44 KH2PO4, 0.18 Na2HPO4, 0.1 EGTA, 25 HEPES, and 5.55 Dextrose (pH 7.3). To quantify ATP release, the BM and SV were incubated in ZCS for 20 min at 37°C, 5% CO2. The collected incubation solutions were kept on ice. The amount of ATP was measured by a bioluminescence method with a luciferin-luciferase assay kit (FL-ASC, Sigma, USA) using a black 96-well plate to avoid optical cross-talk. The bioluminescence was read by a Biotek Synergy 4 Hybrid Microplate Reader (Biotek Instruments Inc, Winooski, VT, USA). All bioluminescence measurements reported in this article fell within the linearity range of the ATP standard curve generated according to the manufacturer’s instructions.

### Measurement of Endocochlear Potential

The endocochlear potential was recorded under general anesthesia on Cx30^−/−^ (*n* = 6) mice and Wt (*n* = 6) mice at 1- month-old. Mice were anesthetized with ketamine (150 mg/kg, IP) and xylazine (6 mg/kg, IP). Body temperature was maintained at 37°C on a heating operating table (Harvard Apparatus, 73-3771). A mouse head holding adaptor (MA-6N, Narishige, Tokyo, Japan) was used to maintain a supine position. A tracheotomy was performed, followed by opening the auditory bulla through a ventral approach to expose the basal turn of the cochlea. A silver chloride reference electrode was placed under the skin. Access to the scala media of the basal turn was obtained by thinning the bone over the spiral ligament and making a small opening with a pick. A micropipette electrode (~2 μm) filled with 150 mM KCl was advanced through the bony aperture into the spiral ligament. Entry of the electrode tip into the endolymph is characterized by transients in recorded potentials. The electrode was advanced until a stable potential was observed. The signal was amplified through a patch-Clamp Amplifier (HEKA EPC 10 USB double, Germany). The DC potentials were recorded *via* an A-D converter (HEKA EPC 10 USB PROBE 1, Germany).

### Transmission Electron Microscope Observation

After the cochlea of P5 mice was dissected, it was fixed in 2.5% glutaraldehyde for 24 h and decalcified with 10% EDTA for several days. The samples were fixed in 1% osmic acid for 2 h, dehydrated with acetone, and embedded in 812 resin. After staining the ultrathin sections with alkaline lead citrate and uranyl acetate, the structure of each cochlea under a Hitachi HF5000 transmission electron microscope (Hitachi, Tokyo, Japan) was observed.

### Statistics

Several statistical methods were used to analyze the data. The Kolmogorov-Smirnov test was used to analyze the normality of the distribution. The Student *t*-test compared statistical means. The Mann-Whitney U test was used for non-normal distributions or distributions with different variances. All statistical analyses were performed using SPSS v19.0 (SPSS Inc. Chicago, IL). Mean values are quoted ± standard error of the mean (s.e.m.) where *p* < 0.05 was assumed as statistically significant.

## Results

### Deletion of Cx30 in the Cochlea

**Figure 1:**
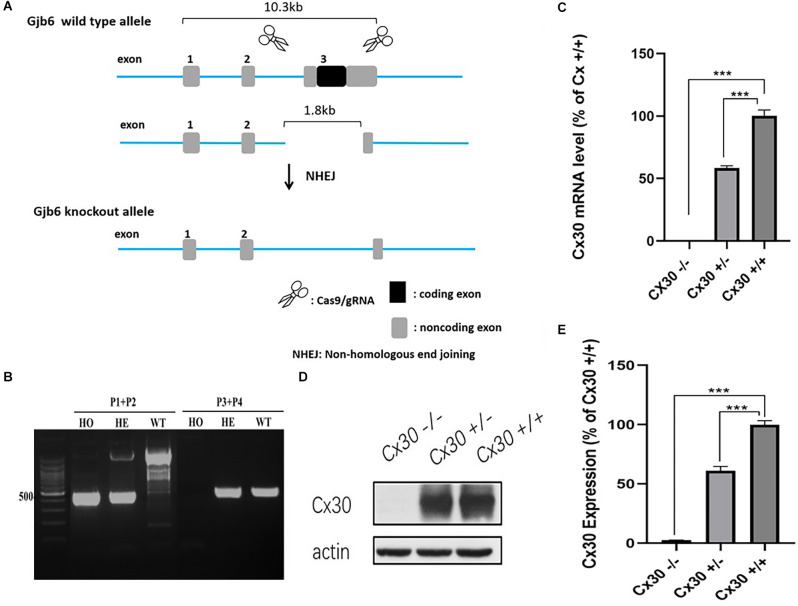
Cx30^−/−^ mice model. **(A)** CRISPR/Cas9 technology was used to repair the mutation introduced by non-homologous recombination, resulting in the shift of the protein reading frame of the *Gjb6* Exon3 gene. **(B)** PCR genotyping: Wild type (WT): *Gjb6* primer 1 (P1) and P2 PCR to obtain a single 2,369 bp band; *Gjb6* P3 and P4 can obtain a 512 bp band; Heterozygous (HE): *Gjb6* P1 and P2 PCR to obtain both 527 bp and 2,369 bp bands; *Gjb6* P3 and P4 to obtain 512 bp band; Homozygous (HO): *Gjb6* P1 and P2 PCR obtained a single 527 bp band; *Gjb6* P3 and P4 could not obtain a band. **(C–E)** Cx30 expression was quantified by qPCR **(C)** and western blot **(D,E)** in the whole cochlea. Data presented as Mean ± SEM; three independent experiments; three mice of each genotype per experiment; *t*-test. ***p < 0.001.

In our model, CRISPR/Cas9 technology was used to repair the mutation introduced by non-homologous recombination, resulting in a shift of the protein reading frame of the *Gjb6* exon 3 gene and loss of function (Figures 1A,B). To test for Cx30 inactivation, we measured Cx30 expression in the inner ear by qPCR (Figure 1C). As expected, no Cx30 transcript was detected in Cx30^−/−^ mice, whereas Cx30^+/–^ mice displayed a 57.6% reduction in expression of Cx30 mRNA compared to Cx30^+/+^ (WT) control mice. Consistent with the mRNA results, Western blot analysis of protein levels in the inner ear of Cx30 +/– mice showed a 61.1% reduction compared to WT mice, while it was undetectable in Cx30 ^−/−^ mice (Figures 1D,E).

### Hearing Loss in Cx30^−/−^ Mice

**Figure 2:**
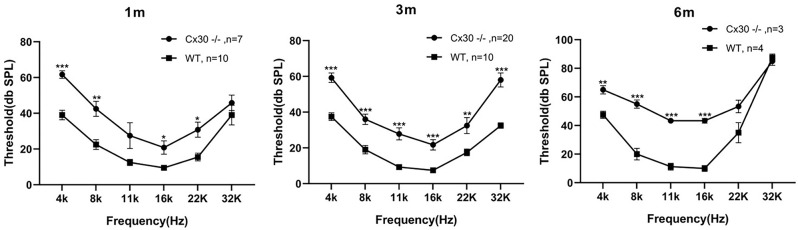
Mild hearing loss in Cx30^−/−^ mice. ABR waveforms were recorded from Cx30^−/−^ and WT littermate mice at 1 m, 3 m, 6 m. 1 m: 1-month-old; 3 m: 3-month-old; 6 m: 6-month-old. Data presented as Mean ± SEM; *n* > 3 in each group. *t*-test.*p < 0.05, **p < 0.01, ***p < 0.001. ABR, Auditory brainstem response.

One-month-old, 3-month-old and 6-month-old Cx30^−/−^ mice displayed mild hearing loss at the full frequency (Figure 2). Compared with WT mice, the ABR thresholds in 1-month-old Cx30^−/−^ mice at 4, 8, 11, 16, 22, 32, and 40 kHz were elevated by 22.7 ± 2.1 (*p* < 0.001), 20 ± 4.2 (*p* < 0.01), 15 ± 5.9, 11.3 ± 3.8 (*p* < 0.01), 15.3 ± 4.2 (*p* < 0.05), and 6.8 ± 4.4 dB SPL, respectively. Compared with WT mice, the ABR thresholds in 3-month-old Cx30^−/−^ mice at 4, 8, 11, 16, 22, 32, and 40 kHz were elevated by 21.8 ± 2.62 (*p* < 0.001), 17 ± 2.9 (*p* < 0.001), 18.6 ± 3.4 (*p* < 0.001), 14.3 ± 2.9 (*p* < 0.001), 15 ± 4.5 (*p* < 0.01), and 25.5 ± 3.9 (*p* < 0.001) dB SPL, respectively. Compared with WT mice, the ABR thresholds in 6-month-old Cx30^−/−^ mice at 4, 8, 11, 16, 22, 32, and 40 kHz were elevated by 17.5 ± 2.9 (*p* < 0.01), 35 ± 2.9 (*p* < 0.001), 32.1 ± 1.7 (*p* < 0.001), 33.3 ± 1.7 (*p* < 0.001), 18.3 ± 4.4, and −2.5 ± 2.9 dB SPL, respectively. At 6-months of age, the high-frequency hearing thresholds in Cx30^−/−^ mice and WT mice were nearly identical.

### Normal Cochlea Microstructure in Cx30^−/−^ Mice

**Figure 3:**
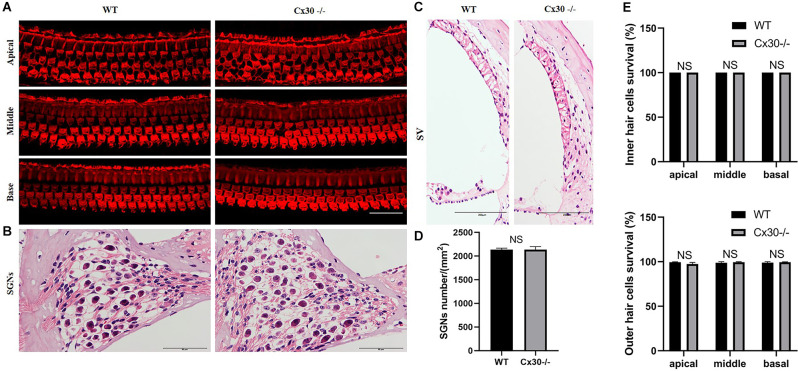
The microscopic structure of cochlea in Cx30^−/−^ mice. **(A,E)** Whole-mountings of the apical, middle and basal turns of cochlear sensory epithelium in 1-month-old Cx30^−/−^ mice and WT mice were obtained by maximal intensity back-projection of 15 confocal optical sections from a 0.8 μm step through—focus sequence (z-stack); actin filaments were stained with phalloidin (red); scale bar: 50 μm. **(B,D)** HE staining of frozen sections of the cochlea in Cx30^−/−^ mice and WT mice showed no difference in the number of spiral ganglions (SGNs). Scale bar: 50 μm. **(C)** HE staining of frozen sections of the cochlea in Cx30^−/−^ mice and WT mice showed no difference in the thickness of SV. SV, stria vascularis. Scale bar: 50 μm. NS, not statistically significant. Data presented as Mean ± SEM. *t*-test.

Cx30^−/−^ mice had no cochlear developmental disorders (Figure 3). Whole-mounting of the apical, middle, and basal turns of the cochlear sensory epithelium of 1-month-old Cx30^−/−^ mice and WT mice showed no apparent hair cell loss (Figures 3A,E). HE staining of cochlear frozen sections in Cx30^−/−^ mice and WT mice showed no difference in the number of SGNs and the thickness of SV (Figures 3B–D).

### Cx26 Expression in Cx30^−/−^ Mice

**Figure 4:**
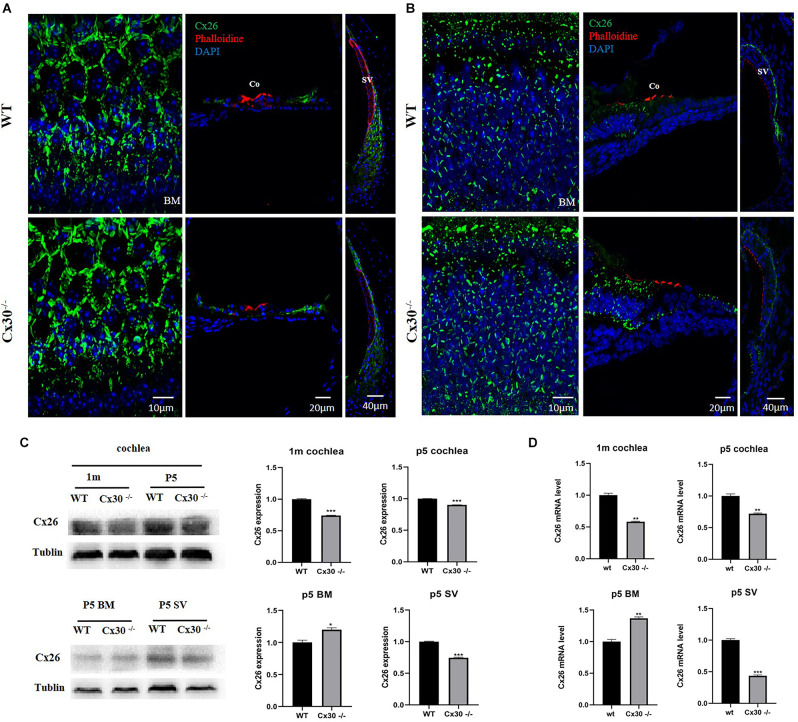
Cx26 expression in Cx30^−/−^ mice. **(A)** Immunofluorescence analysis of frozen sections and BM in the cochlea of 1-month-old mouse. **(B)** Immunofluorescence analysis of frozen sections and BM in the cochlea at P5. Both in 1-month-old and P5, Cx26 expression in the BM of Cx30^−/−^ mice was stronger than that of WT mice, and Cx26 expression in the vascular stripe of Cx30^−/−^ mice was significantly lower than that of WT mice. Cx26: Green, Phalloidin: Red, DAPI: Blue (for cell nuclei); scale bar: 10 μm, 20 μm, 40 μm. **(C)** Western blot analysis of the cochlea, BM, and SV in 1-month-old and P5 mice. Whole cochlear Cx26 protein levels in 1-month-old Cx30^−/−^ mice showed a 25.9% decrease compared with WT mice; whole cochlear Cx26 protein levels of Cx30^−/−^ mice showed 9.84% reduction compared with WT mice at P5; cochlear BM Cx26 protein levels of Cx30^−/−^ mice increased by 19.8% compared with WT mice at P5; Cx26 protein level in SV of the cochlea in Cx30^−/−^ mice decreased by 25.6% compared with WT mice in P5. **(D)** qPCR analysis of the cochlea, BM, and SV in 1-month-old and P5 mice. Whole cochlear Cx26 mRNA levels of 1-month-old Cx30^−/−^ mice declined by 41.8% compared with WT mice; whole cochlear mRNA levels of Cx30^−/−^ mice decreased by 28% compared with WT mice at P5; cochlear BM Cx26 mRNA levels in Cx30^−/−^ mice increased by 36.7% compared with WT mice at P5; cochlear SV Cx26 mRNA levels in Cx30^−/−^ mice reduced by 56.6% compared with WT mice at P5. Data presented as Mean ± SEM. *t*-test. *p < 0.05, **p < 0.01, ***p < 0.001. BM, basemental membrane; SV, stria vascularis; Co, Corti’s organ.

Immunofluorescence showed that Cx26 expression in the BM of Cx30^−/−^ mice was stronger than that of WT mice, and Cx26 expression in the vascular stripe of Cx30^−/−^ mice was significantly lower than that of WT mice both in 1-mouth-old and P5 (Figures 4A,B).The mRNA and protein levels of Cx26 in the cochlea of Cx30^−/−^ mice relative to that of WT mice were quantified (Figures 4C,D). qPCR analysis showed that the whole cochlear Cx26 mRNA levels of 1-month-old Cx30^−/−^ mice decreased by 41.8% compared with WT mice; whole cochlear mRNA levels in Cx30^−/−^ mice decreased by 28% compared with WT mice at P5; cochlear BM Cx26 mRNA levels in Cx30^−/−^ mice increased by 36.7% compared with WT mice at P5; cochlear SV Cx26 mRNA levels in Cx30^−/−^ mice decreased by 56.6% compared with WT mice in P5 (Figure 4D). Western blot showed that the whole cochlear Cx26 protein levels in 1-month-old Cx30^−/−^ mice decreased by 25.9% compared with WT mice; whole cochlear Cx26 protein levels of Cx30^−/−^ mice reduced by 9.84% compared with WT mice at P5; cochlear BM Cx26 protein levels of Cx30^−/−^ mice increased by 19.8% compared with WT mice at P5; cochlear SV Cx26 protein level of Cx30^−/−^ mice decreased by 25.6% compared with WT mice in P5 (Figure 4C).

### Mild Damaged Stria Vascularis Submicroscopic Structure in Cx30^−/−^ Mice

**Figure 5:**
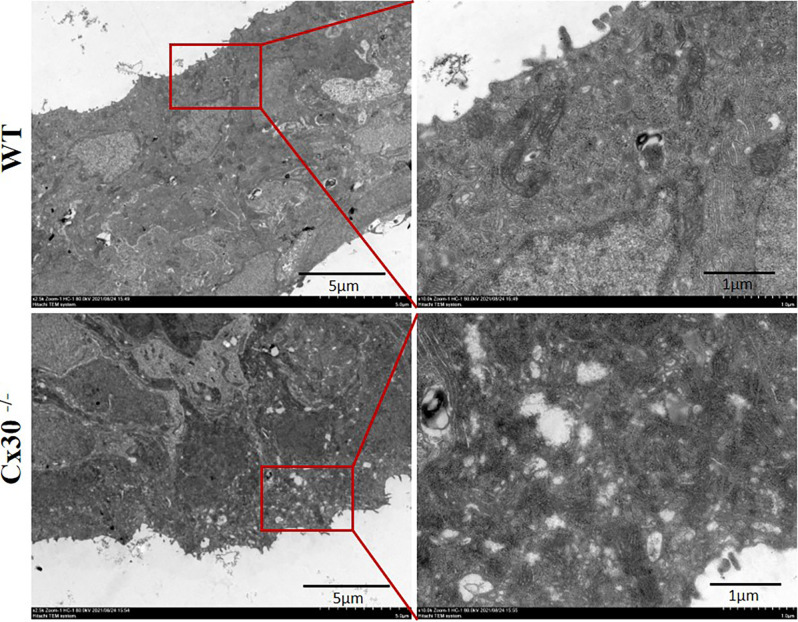
Transmission electron microscopy of the submicroscopic structure of the stria vascularis in Cx30^−/−^ mice. Visible cavity-like damage in the SV of Cx30^−/−^ mice compared with WT mice. Scale bar: 5 μm, 1 μm.

Figure 5 shows the transmission electron microscopy images of the submicroscopic structure of the SV in Cx30^−/−^ mice. There was some visible vacuolar injury in the SV of Cx30^−/−^ mice compared with WT mice, which suggested that Cx30 deletion might cause mild damage to the SV.

### Reduction of Endocochlear Potential in Cx30^−/−^ Mice

**Figure 6:**
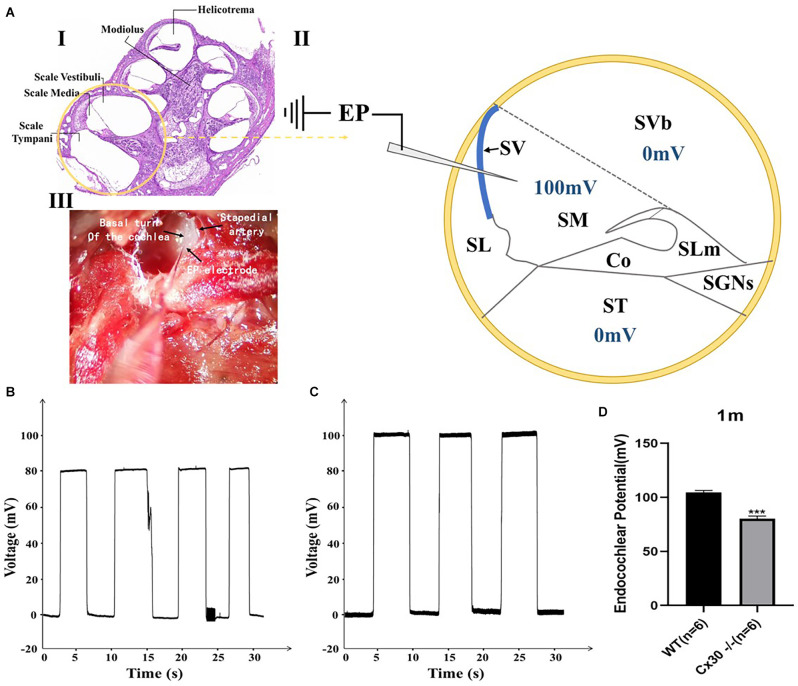
Endocochlear potential (EP) measurements in WT and Cx30 KO mice. **(A)** (I) Cross-section of a cochlea. (II) Schematic diagram of the cross-section of the basal cochlear turn illustrating the method of EP measurement. A microelectrode filled with 3 M KCl was inserted into the endolymph-filled scala media (100 mV in a WT mouse) to record EP. (III) Photograph of a surgically exposed cochlea before EP measurement showing the location of the stapedial artery and the insertion site of the microelectrode. **(B,C)** Representative EP recordings from 1-month-old WT and Cx30 KO mice. The traces showed the voltage (mV) of the scala media as a function of time (s). **(D)** The graph illustrates a significant 23.4% reduction in the EP in 1-month-old Cx30 KO mice (80.1 ± 2.6 mV) compared with age-matched WT mice (104.6 ± 1.9 mV). Data presented as Mean ± SEM (*n* = 6). *t*-test. ***p < 0.001. Co, Corti’s organ; SGNs, spinal ganglion neurons; SL, spiral ligament; SLm, spiral limbus; SM, scala media; ST, scala tympani; SV, stria vascularis; SVb, scala vestibuli.

Figure 6 shows a reduction of EP in 1-month-old Cx30^−/−^ mice compared with the WT mice. EPs in Cx30^−/−^ mice and WT mice were 80.1 ± 2.6 mV and 104.6 ± 1.9 mV, respectively (Figure 6B). EP in Cx30^−/−^ mice was significantly reduced by 23.4% (*p* < 0.001) relative to WT mice.

### Reduction of ATP Release in Cx30^−/−^ Mice

**Figure 7:**
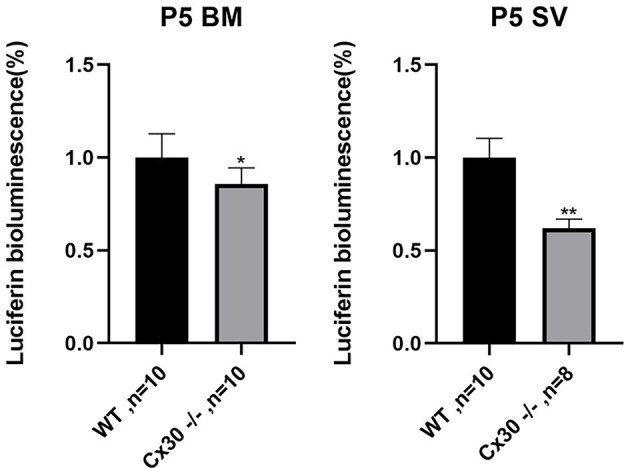
Reduction of ATP release in the cochlea in Cx30^−/−^ mice. ATP release is reduced in Cx30^−/−^ mice in BM and SV at P5. Data presented as Mean ± SEM. *t*-test. *p < 0.05, **p < 0.01. BM, basemental membrane; SV, stria vascularis.

Figure 7 shows reductions in ATP release of Cx30^−/−^ mice relative to WT mice, both in BM and SV at P5. Compared with WT mice, ATP release in Cx30^−/−^ mice was significantly reduced by 14.2% (*p* < 0.05) in BM and 37.9% (*p* < 0.01) in SV, respectively. ATP release of SV decreased more significantly than that of BM.

## Discussion

Seven large genomic deletions of *GJB6* have been previously reported, including >920 kb deletion (Feldmann et al., 2009), 179 kb deletion (Tayoun et al., 2016), 131 kb deletion (Wilch et al., 2010), del(*GJB6*-D13S175), del(*GJB6*-D13S1830), del(*GJB6*-D13S1854; Bliznetz et al., 2017) and del(*GJB6*-D13S1834; Pandya et al., 2020). Homozygous deletion of *GJB6* results in severe to profound hearing loss in humans, similar to the mouse model (Teubner et al., 2003; Mei et al., 2017; Pandya et al., 2020). In many cases, the large deletions of *GJB6* disrupt a 50 cis-acting element upstream of both genes, which abolishes the expression of *GJB2*, and hence is responsible for the phenotype (Rodriguez-Paris and Schrijver, 2009). A recent study reported the del (*GJB6*-D13S1830) deletion in 16.35% of all *GJB2* heterozygotes, which causes more profound hearing loss than those with bi-allelic change in *GJB2* (Pandya et al., 2003, 2020; Snoeckx et al., 2005). The more severe audiological findings in digenic probands cannot be explained by the sole theory of a putative cis-regulatory element in the deleted region (Pandya et al., 2020), suggesting that both *GJB6* and *GJB2* products may contribute to the hearing loss.

The Corti’s organ is the core in the auditory system, which is composed of HCs and SCs. SCs release ATP via connexin which triggers intercellular Ca^2+^ wave propagation and might be critical for the functional maturation of HCs (Johnson et al., 2017). The function of HCs is to transduce the sound mechanical stimulation into the primary acoustic signals (Abeytunge et al., 2021), while the spiral ganglion neurons (SGNs) transmit primary acoustic information from HCs in the organ of Corti to the higher auditory centers of the central nervous system (Wei et al., 2021). *GJB6* and *GJB2* are located on the same chromosome (13q12.11) in the human genome. Due to the relative proximity of these two genes, their expressions might be controlled by the same set of regulatory elements (Wilch et al., 2006). Homozygous deletion mutations of either Cx26 or Cx30 could cause deafness in humans (Kelsell et al., 1997; del Castillo et al., 2002). Cx26 plays a critical role in auditory function and its deficiency leads to significant hearing loss in the animal model (Cohen-Salmon et al., 2002; Chen et al., 2014). Cx26 deficiency in the cochlea could cause pathological changes, including cochlear developmental disorders (Wang et al., 2009; Liang et al., 2012), HCs and SGNs degeneration (Cohen-Salmon et al., 2002; Sun et al., 2009; Wang et al., 2009), EP reduction (Chen et al., 2014), and impairment of active cochlear amplification (Zhu et al., 2013, 2015). HCs and SGNs degeneration was detected around P14 in Cx26-deficient mice (Sun et al., 2009; Wang et al., 2009). However, severe hearing loss precedes substantial hair cell loss after the deletion of Cx26 (Liang et al., 2012). Nevertheless, EP remained normal after targeted deletion of Cx26 expression in Deiters SCs and outer pillar SCs, which are located around OHCs in the cochlear sensory epithelium (Zhu et al., 2013). This indicates that impairment of the cochlear sensory GJ network may not affect EP generation in the inner ear (Zhu et al., 2013; Chen and Zhao, 2014; Chen et al., 2015). However, Cx26 deficiency in the cochlear SCs can affect OHC electromotility and active cochlear amplification, since distortion product otoacoustic emission (DPOAE) was reduced (Yu and Zhao, 2009; Zhu et al., 2013, 2015). On the other hand, the cochlear tunnel opens from P5 (Kraus and Aulbach-Kraus, 1981). Cx26 deficiency before P5 can lead to occlusion of the cochlear tunnel and induce cochlear developmental disorders with congenital deafness (Chen et al., 2014). Nevertheless, cochlear development proceeded normally, the cochlear tunnel opened normally, and hearing remained normal at a young age after deletion of Cx26 after P5 (Chen et al., 2014). These findings suggest that Cx26 expression in the cochlea at the early postnatal development stage is critical for cochlear postnatal development and maturation.

Teubner et al. (2003) found that Cx30 knockout mice display severe hearing loss with the absence of EP. Whether the Cx30 deficiency itself or the accompanying significant decrease of Cx26 (approximately 90%) leads to hearing loss in this model remains unknown. Restoration of Cx26 protein level in the cochlea completely rescues hearing in Cx30 knockout mice, whereas hearing loss in the conditional Cx26 (cCx26) null mice could not be rescued by genetically over-expressing Cx30 (Ahmad et al., 2007; Qu et al., 2012). Cx26 protein expression preceded that of Cx30, and Cx26 is the only and essential GJ protein detected by immunolabeling in the organ of Corti during the early postnatal period (Qu et al., 2012). During the postnatal development, the lack of GJs in the Corti’s organ leads to obstacles in intercellular material transport and communication, and failure of Corti’s organ to mature, leading to hearing loss. However, the Cx26 expression of BM increased in Cx30^−/−^ mice at the early postnatal period (P5) in the present study.

Boulay et al. (2013) established Cx30^fl/fl^ mice, and found no significant difference in hearing between Cx30^fl/fl^ mice and WT mice, however, Cx26 was decreased to 35% and Cx30 was reduced to 64%. Normal EP and DPOAE were observed in this kind of mice (Boulay et al., 2013). Crossing Cx30^fl/fl^ mice with Pgk-Cre mice generated Cx30^Δ/Δ^ mice to delete Cx30, which exhibited no hearing loss, while Cx26 was decreased to 52% (Boulay et al., 2013). The threshold level of Cx26 decline in Cx30-deficient mice that causes hearing loss remains unclear. The Cx30^−/−^ mice established in this study exhibited only mild hearing loss, while cochlear Cx26 decreased to 74%. Since Cx26 did not show any major decrease through Cx30 knockout, the mechanism of hearing loss needs to be further explored.

The high positive potential of EP is critical for animal hearing and develops rapidly in the days before the onset of hearing (P4–P11; Chen et al., 2015). EP (+100–110 mV) is the driving force for the generation of auditory receptor currents and potentials by K^+^ ions through transduction channels in the HCs, which are formed by a complex process in the SV of the cochlea. The widely accepted “two-cell” model suggests that EP production begins in the fibroblasts of the spiral ligament. Type II fibroblasts depolarize the cells to ~5 mV *via* Na^+^/K^+^ - ATPase and Na^+^, K^+^, 2Cl^−^ cotransport proteins. The intermediate cells of the SV are subsequently coupled by Cx26 and Cx30 *via* GJs. Thereafter, the ATP-dependent Kir4.1 K^+^ channel on the apical membrane of intermediate cells (Ando and Takeuchi, 1999; Liu and Zhao, 2008; Nin et al., 2008) generates a 105–110 mV transmembrane potential (Nernst’s K^ +^ balance potential) between the intracellular space and the intrastriatal space. This positive intrastriatal potential eventually leads to EP positivity in the middle-grade endolymph. The network of two GJs composed of Cx26 and Cx30 forms a pathway through which K^+^ ions that pass through sensory cells during mechanosensory transmission can be recirculated into the endolymphatic space such that they re-enter sensory cells (Wangemann, 2002). GJs deficiency may lead to the obstruction of K^+^ circulation, thus affecting the formation of EP and hearing. ATP is necessary for EP generation and K^+^ recycling (Zhu and Zhao, 2010; Chen et al., 2015). The perfusion of ATP into the cochlea can significantly increase EP (Sueta et al., 2003). As described above, depolarization of fibroblasts by co-activation of Na^ +^/K^ +^ - ATPase and Na^ +^, K^ +^, 2Cl^−^-cotransporters is the first step in EP production. Although Na^+^/K^+^ - ATPase is primarily driven by intracellular ATP, extracellular ATP can stimulate Na^+^/K^+^ - ATPase activity by activating purinergic receptor and Src family kinase (SFK) as well (Shahidullah et al., 2012). In addition, the function of the primary active Na^+^/K^+^ - ATPase requires K-channel coupling to recycle K^+^ (Muto et al., 2003). This “pump coupling” was proposed in 1958, and was subsequently confirmed by experimental data (Koefoed-Johnsen and Ussing, 1958; Dawson and Richards, 1990; Tsuchiya et al., 1992). Recently, it was found that extracellular ATP can also activate ATP-sensitive (KIR) K channels in hippocampal CA3 pyramidal neurons and lung epithelial cells, which cooperate with Na^+^/K^+^ - ATPase activity. ATP may initially activate the P2X receptor and subsequently activate the KIR potassium channel and Na^+^/K^+^ - ATPase (Telang et al., 2010; Jiang et al., 2013; Schmid and Evans, 2019).

In this study, we established a novel Cx30 complete knockout mouse model, with a mild full-frequency hearing loss as indicated by ABR. The protein level of Cx26 was decreased by less than 30% in the cochlea of these mice, which was significantly different from the previous Cx30-deficient mouse model. Moreover, both EP and ATP release showed a decreasing trend in the present mouse model. These results suggest that Cx30 may play an important role in hearing development, however, further investigation on the underlying mechanism is needed in the future.

## Data Availability Statement

The original contributions presented in the study are included in the article, further inquiries can be directed to the corresponding author/s.

## Ethics Statement

The animal study was reviewed and approved by the Ethics Committee of Xin Hua Hospital Affiliated to Shanghai Jiao Tong University School of Medicine (XHEC-2021-636).

## Author Contributions

JC: experimental implementation, data quality control, and wrote the manuscript. PC: immunohistochemical staining and statistical data analysis. BH: western blot and qPCR. TG: qPCR experiments. YL: collection of cochlear samples for TEM experiments. JZ: collection of cochlear samples for H&E experiments. JL: collection of cochlear samples for TEM experiments. FM: EP experiments. SH: research conception and experimental supervision. JY: research conception, manuscript review and revision. All authors contributed to the article and approved the submitted version.

## Conflict of Interest

The authors declare that the research was conducted in the absence of any commercial or financial relationships that could be construed as a potential conflict of interest.

## Publisher’s Note

All claims expressed in this article are solely those of the authors and do not necessarily represent those of their affiliated organizations, or those of the publisher, the editors and the reviewers. Any product that may be evaluated in this article, or claim that may be made by its manufacturer, is not guaranteed or endorsed by the publisher.
